# Genome Sequence of *Actinobacillus suis* Type Strain ATCC 33415^T^

**DOI:** 10.1128/genomeA.00926-14

**Published:** 2014-09-18

**Authors:** Michael J. Calcutt, Mark F. Foecking, Tendai Mhlanga-Mutangadura, Thomas J. Reilly

**Affiliations:** a Department of Veterinary Pathobiology, University of Missouri, Columbia, Missouri, USA; b Veterinary Medical Diagnostic Laboratory, Department of Veterinary Pathobiology, University of Missouri, Columbia, Missouri, USA

## Abstract

The assembled and annotated genome of *Actinobacillus suis* ATCC 33415^T^ is reported here. The 2,501,598-bp genome encodes 2,246 open reading frames (ORFs) with strain variable incursion of an integrative conjugative element into a tRNA locus. Comparative analysis of the deduced gene set should inform our understanding of pathogenesis, genomic plasticity, and serotype variation.

## GENOME ANNOUNCEMENT

Among the infectious agents that are emerging challenges to high-health-status swine herds, *Actinobacillus suis* is of particular importance, causing septicemia with significant mortality in younger animals (1). Although a number of putative virulence genes have been identified (2–4), whole-genome sequencing of the type strain, isolated as strain 1276/61 from a septicemic piglet (5), was undertaken to more completely assess the gene repertoire of this pathogen. While this project was under way, the genome sequence of the *A. suis* serotype O2 isolate H91-0380 was determined (6).

Titanium fragment library sequencing of genomic DNA from *A. suis* ATCC 33415^T^ (obtained directly from ATCC) was performed using the 454 platform at The Genome Institute, Washington University, St. Louis, MO. Following Newbler assembly of 296,239 reads, 31 large contigs (>500 bp) were obtained with 52-fold genome coverage. Guided by an optical map of AflII restriction fragments (generated by Opgen, Gaithersburg, MD), gaps were closed by PCR amplification and amplicon sequencing with BigDye terminator Sanger chemistry. The sequence of a short intergenic region containing an extended homopolymeric tract proved refractory to accurate determination by either 454 or Sanger methodologies. The final assembly was autoannotated using the PGAP pipeline at the NCBI prior to validation and manual curation of 11 pseudogenes with deletions, frameshift mutations, or premature stop codons. The small stable RNA-encoding genes *rnpB* and *ssrA* were identified through similarity to matches from *Actinobacillus pleuropneumoniae* in the ribonuclease P (7) and transfer-messenger RNA (tmRNA) (8) databases.

The single, 2,501,598-bp circular chromosome has a G+C content of 40.22% and encodes 2,341 genes, comprising 2,246 open reading frames (ORFs), 63 tRNAs, and 19 rRNAs (1 of 6 *rrn* operons contained an additional *rrf* gene). Most ORFs have counterparts in the 16,658-bp-smaller H91-0380 genome, with complete synteny between regions present in both genomes. The two largest regions of difference result from variations in mobile genetic element portfolios of the respective mobilomes. Whereas strain H91-0380 contains two prophage genomes, only one of these (~15 kb) is present in ATCC 33415^T^. The latter strain contains an additional 55,589-bp locus that resembles a strain-variable integrative conjugative element of *A. pleuropneumoniae* serotype 6 strain Femo; this element is inserted into an orthologous Leu tRNA-encoding gene in each isolate. A single clustered regularly interspaced short palindromic repeat (CRISPR) locus was detected (9) with 15 spacers of 30 bp and 16 direct repeats of 36-bp length. Eight of the spacers matched equivalently ordered units in the array encoded within H91-0380, and identical CRISPR repeats were identified in 7 additional species from within the *Pasteurellaceae*.

With the availability of genome sequences for strains of the two predominant O antigen serotypes (10), comparative genomic analyses are under way to assess the pangenome of the species, the possible consequences of strain variable loci, and the role that lateral gene transfer has played in shaping the core and accessory genomes. These data will be a resource for the future postgenomic study of species evolution, pathogenesis, and vaccine development.

### Nucleotide sequence accession number.

This complete genome sequence has been deposited at DDBJ/EMBL/GenBank under the accession number CP009159.

## References

[1] MacInnes JI Desrosiers R 1999 Agents of the “suis-ide diseases” of swine: *Actinobacillus suis*, *Haemophilus parasuis*, and *Streptococcus suis*. Can. J. Vet. Res. 63:83–89 10369563 PMC1189524

[2] Burrows LL Lo RY 1992 Molecular characterization of an RTX toxin determinant from *Actinobacillus suis*. Infect. Immun. 60:2166–2173 1587585 10.1128/iai.60.6.2166-2173.1992 PMC257139

[3] Bahrami F Ekins A Niven DF 2003 Iron acquisition by *Actinobacillus suis*: identification and characterization of transferrin receptor proteins and encoding genes. Vet. Microbiol. 94:79–92. 10.1016/S0378-1135(03)00082-8 12742718

[4] Ojha S Lacouture S Gottschalk M MacInnes JI 2010 Characterization of colonization-deficient mutants of *Actinobacillus suis*. Vet. Microbiol. 140:122–130. 10.1016/j.vetmic.2009.07.014 19664889

[5] VanDorssen CA Jaartsveld FJH 1962 *Actinobacillus suis* (novo species), een bij het varken voorkomende bacterie. Tijdschr. Diergenbeeskd. 87:450–458

[6] MacInnes JI Mackinnon J Bujold AR Ziebell K Kropinski AM Nash JH 2012 Complete genome sequence of *Actinobacillus suis* H91-0380, a virulent serotype O2 strain. J. Bacteriol. 194:6686–6687. 10.1128/JB.01633-12 23144422 PMC3497502

[7] Brown JW 1997 The ribonuclease P database. Nucleic Acids Res. 27:314. 10.1093/nar/27.1.314 9847214 PMC148169

[8] Gueneau de Novoa P Williams KP 2004 The tmRNA website: reductive evolution of tmRNA in plastids and other endosymbionts. Nucleic Acids Res. 32(Suppl 1):D104–D108. 10.1093/nar/gkh102 14681369 PMC308836

[9] Grissa I Vergnaud G Pourcel C 2007 CRISPRFinder: a Web tool to identify clustered regularly interspaced short palindromic repeats. Nucleic Acids Res. 35(Suppl 2):W52–W57. 10.1093/nar/gkm360 17537822 PMC1933234

[10] Slavic D Toffner TL Monteiro MA Perry MB MacInnes JI 2000 Prevalence of O1/K1- and O2/K3-reactive *Actinobacillus suis* in healthy and diseased swine. J. Clin. Microbiol. 38:3759–3762 11015398 10.1128/jcm.38.10.3759-3762.2000 PMC87471

